# 635. Multi-staged Participatory Co-design of a Childhood Burns Manual for Village Health Teams in Ugandan Slums

**DOI:** 10.1093/jbcr/irag033.461

**Published:** 2026-04-06

**Authors:** Alys Barton, Shweta Gidwani, Charles Ssemugabo, Jimmy Osuret, Marcia Tusiime, Dennis Mazingi, Alejandra Piragauta, Prasanthi Attwood, Margie Peden, Julie Brown

**Affiliations:** George Washington University, Washington, DC; The George Institute for Global Health UK / Imperial College London, UK, London, England; Makerere University, Kampala, Uganda; Makerere University, Kampala, Uganda; Makerere University, Kampala, Uganda; The George Institute for Global Health UK, London, England; The George Institute for Global Health UK, London, England; The George Institute for Global Health UK, London, England; The George Institute for Global Health UK, London, England; The George Institute for Global Health Australia, Sydney, NSW

## Abstract

**Introduction:**

Global burn related deaths are common in children under 5 years and are disproportionately high prevalence in low-to-middle income countries. In urban slum areas of Kampala, Uganda, burns are the leading cause of injury in children under five. Children are at increased risk of burn injuries when living in slum communities due to exposure to scald, contact and flame burns from traditional cooking methods. Evidence-based practice recommends immediate cooling of burns with clean running water however in the community, common practice involves using unconventional substances such as eggs, sugar, toothpaste, urine, cow dung and motor oil. The objective of this participatory study was to co-design a culturally and contextually appropriate training manual for village health teams (VHTs) that will empower them to perform and demonstrate appropriate response and first aid for burns in the community.

**Methods:**

We conducted a multi-staged participatory study from March 2024 to October 2024. A childhood burns first aid training curriculum was co-designed for VHTs in three stages. In the first stage, we identified the gaps in burn training and response with preliminary studies and a comprehensive literature review to inform a prototype. In the second stage, we conducted iterative virtual workshops with mapped stakeholders to understand learning needs, assess delivery methods and integrate feedback into successive versions of a more relevant training manual. In the third stage, we held in-person workshops for VHTs and subject matter experts to pilot the material, assess acceptability and share key contributions to the final burn training manual.

**Results:**

A community first response burns care training manual was disseminated in English and Luganda to VHTs. All workshop participants were acknowledged by name for their contributions.

**Conclusions:**

The participatory approach to co-designing a training package enabled stakeholder engagement and community ownership of the material. This train-the trainer model for effective burns first aid should be evaluated for its content validity and feasibility of roll out in future studies.

**Applicability of Research to Practice:**

The multi-staged participatory co-design approach resulted in a scalable framework that can be applied to unique settings and target populations with high burn burden or other public health issues.

**Funding for the study:**

This study was funded by the International Science Partnerships Fund (ISPF) through Research England.

